# Prevalence, comorbidity, and demographic patterns of oppositional defiant disorder and conduct disorder in Chinese children and adolescents: a systematic review

**DOI:** 10.3389/fpsyg.2025.1691623

**Published:** 2026-01-21

**Authors:** S. Mudasser Shah, Ghada Saleh Alhudaithi, Ghada Saad Altalha, Chand Taneja, Fatimah Sayer Alharbi, Xiuyun Lin

**Affiliations:** 1 Institute of Developmental Psychology, Beijing Normal University, Beijing, China; 2 Self-Development Skills Department of common First Year Deanship, King Saud University, Riyadh, Saudi Arabia; 3 Department of Self-Development Skills, King Saud University, Riyadh, Saudi Arabia; 4 Department of Neuropsychology Program, Queen Alexandra Center for Children’s Health, Victoria, BC, Canada; 5 Department of Psychology, College of Education and Human Development, Princess Nourah Bint Abdulrahman University, Riyadh, Saudi Arabia; 6 Beijing Key Laboratory of Applied Experimental Psychology, Faculty of Psychology, Beijing Normal University, Beijing, China

**Keywords:** oppositional defiant disorder, conduct disorder, children, adolescents, China, prevalence

## Abstract

**Introduction:**

The prevalence of major disruptive behavior disorders such as oppositional defiant disorder (ODD) and conduct disorder (CD) has not been fully described in Chinese youth, and their respective patterns are also understudied. This systematic review was conducted to explore prevalence rates, comorbidity, and demographic characteristics of ODD and CD in Chinese children and adolescents.

**Methods:**

A thorough search of international databases revealed 19 peer-reviewed studies published in English between 2016 and 2025 that were pertinent and met the eligibility standards. The findings indicate that the prevalence rates of ODD and CD in Chinese youth were mostly lower than global rates; however, there was comorbidity with attention-deficit/hyperactivity disorder and depressive symptoms.

**Results:**

In several studies, it was noted that boys had higher rates of disruptive behaviors compared to girls, and urban–rural differences influenced diagnosis rates. Parenting practices, peer rejection, and family conflict were identified as predictors of symptom persistence through longitudinal studies, while deficiencies in executive functioning and emotional regulation were noted in neurocognitive research as significant interpersonal pathways. The use of advanced analytic methods, such as structural equation modeling and cross-lagged designs, strengthened causal inferences; however, comparability was restricted by methodological heterogeneity.

**Conclusion:**

The prevalence of cross-sectional studies and reliance on parent/teacher reports limited conclusions. Future studies are encouraged to utilize culturally adapted diagnostic measures, longitudinal designs, and interventional approaches. The results highlight the importance of culturally responsive prevention and treatment interventions focused on the Chinese educational and family contexts.

## Introduction

Oppositional defiant disorder (odd) and conduct disorder (CD) are classified as disruptive behavior disorders in the DSM-5-TR, linked to considerable emotional, academic, and social difficulties among youth (Demmer et al., 2017). Global epidemiological studies indicate significant variability in the prevalence of these disorders, with ODD estimated at 2–11% and CD at 3–9%, establishing a crucial global framework for comparison (American Academy of Family Physicians, 2016; Ayano et al., 2024). Research conducted in Western nations, including the United States and Norway, indicates ODD prevalence rates of approximately 3.3%, accompanied by marginally lower yet frequently comorbid CD rates (Field, 2024). Placing this in a global context helps us understand how cultural and diagnostic differences affect the prevalence of these disorders (Chen et al., 2020).

Chinese epidemiological studies reveal significant differences by region, with ODD rates fluctuating between 1.9 and 8.6%, and CD rates ranging from 1.4 to 6.3% (Liu et al., 2025; Wang et al., 2022; Deng et al., 2023). According to national survey data, 17.5% of children aged 6 to 16 have a psychiatric disorder, with ODD being one of the most common (Li et al., 2022). However, these findings are fragmented across provinces and employ inconsistent methodologies, complicating the interpretation of national estimates. Prior systematic reviews, including either focus solely on CD or omit cultural analysis, thereby limiting their effectiveness in elucidating Chinese-specific patterns. Recent studies indicate that the appearance and severity of ODD and CD in China may be influenced by parenting practices, family dynamics, and overarching sociocultural trends. Urban–rural disparities impact risk variably: urban youth frequently face academic stress and diminished parental oversight, while rural children may encounter parental migration, disrupted caregiving, and limited access to mental health services. Family-level stressors, including authoritarian parenting, parental mental health issues, and intergenerational separation, continue to be substantial risk factors in both China and Chinese diaspora communities (Tang et al., 2017; Zhang et al., 2023). Research from Iran and South Korea indicates that collectivist cultural values may inhibit overt oppositional behavior, leading to the underrecognition of symptoms (Ghaitasi and Amani, 2025), potentially reflecting similar trends observed in China.

Western literature identifies neuropsychological characteristics, challenges in emotional regulation, and academic-related stress as significant factors contributing to both disorders (Bonham et al., 2021; Njardvik et al., 2025; Elbagir et al., 2023). Asian cultural norms that emphasize obedience and emotional restraint may affect how individuals express or understand symptoms of ODD and CD, which could influence their identification and treatment. Comorbidity with conditions such as ADHD, ASD, and depression further complicates diagnosis (Deng et al., 2023; Ding et al., 2019; Ding et al., 2020; Ding et al., 2022). If ODD and CD are not treated, the likelihood of dropping out of school and later engaging in antisocial behavior increases (Demmer et al., 2017). Despite the growing number of intervention studies in China, most available treatments are based on Western models and fail to consider cultural values, family structures, or the pressures of school.

Comorbidity with conditions such as autism spectrum disorder (ASD), ADHD, depression, and anxiety is also common, complicating diagnosis and treatment (Deng et al., 2023; Njardvik et al., 2025; Chou et al., 2019. Both Western and Asian studies note that untreated ODD and CD increase the risk of school dropout, delinquency, and persistent antisocial behavior (Demmer et al., 2017; Xie et al., 2024), highlighting the need for culturally informed, evidence-based interventions. Thus far, most treatments for ODD and CD are based on Western theoretical perspectives (Fairchild et al., 2019; Burke et al., 2002), but cultural differences cannot be ignored. In China, these disorders are influenced by urbanization, educational pressures, and family changes, including those resulting from the One-Child Policy (Wan et al., 2023; Liu et al., 2024). Adherence to Confucian values, such as respect for elders, may inhibit the expression of symptoms, complicating early identification (Wang et al., 2024; Finlay-Jones et al., 2024). Although research on interventions for disruptive disorders in youth is increasing, the literature remains fragmented and often lacks integration of neurodevelopmental and sociocultural factors (Lin et al., 2022). Therefore, a systematic review is needed to synthesize current findings, refine culturally appropriate assessment methods, and support evidence-based, culturally sensitive care for children and adolescents in China.

Given the fragmented nature of Chinese evidence, the limitations of previous systematic reviews, and the absence of a culturally grounded synthesis, a new systematic review is necessary to integrate prevalence data, evaluate methodological quality, and guide culturally informed assessment and intervention for ODD and CD in Chinese youth. Therefore, the present study systematically reviewed and synthesized existing evidence on ODD and CD among Chinese children and adolescents. Specifically, the review aimed to identify prevalence rates, examine patterns of comorbidity with other psychiatric disorders such as ADHD and depression, and explore demographic variations including age, gender, and urban–rural differences. By situating Chinese findings within the broader international literature, the study aimed to highlight cultural, familial, and neurocognitive influences shaping ODD and CD, thereby informing contextually appropriate diagnosis, prevention, and intervention strategies.

## Methodology

The Preferred Reporting Items for Systematic Reviews and Meta-Analyses (PRISMA) guidelines were used to conduct the review, ensuring clarity, scientific robustness, and reproducibility. To synthesize existing empirical literature regarding the prevalence of ODD and CD in Chinese children and adolescents, the main goals were to identify contributing factors to both disorders and to highlight similarities and differences between them in this population. A standard search strategy was implemented to locate peer-reviewed articles in several research databases, including PubMed, PsycINFO, Web of Science, EMBASE, CINAHL, and ERIC. Additionally, searches were conducted in online databases, and reference lists were checked manually. A total of 703 records were initially identified in this comprehensive process. Subsequently, duplicates were removed, leaving 129 unique articles for screening. Titles and abstracts allowed us to exclude 64 studies that were not related to ODD/CD or adult populations and did not pertain to the Chinese context. Of these, 65 full-text articles were evaluated for eligibility. Out of those, 46 were eliminated: 23 due to population issues (e.g., incorrect population type, such as non-child or non-Chinese samples) and 23 for not being peer-reviewed. Finally, 19 studies were retained in the qualitative synthesis that satisfied all inclusion criteria.

### Literature search strategy

A comprehensive search using specific keywords was conducted across five major databases PubMed, PsycINFO, Web of Science, Scopus, and EMBASE, from January 2015 to April 2025. The strategy combined controlled vocabulary (e.g., MeSH) and free-text keywords for terms such as “Oppositional Defiant Disorder,” “Conduct Disorder,” “children,” “adolescents,” and “China.” Boolean operators (AND/OR) were used to maximize both accuracy and sensitivity. The search strategy was tailored for each database, and citation tracking was performed on eligible full-text documents to ensure all relevant studies were included.

### Study selection process

Two independent reviewers screened the titles and abstracts of the identified citations. Articles meeting eligibility criteria were reviewed in detail at the full-text stage. Disagreements were resolved through discussion, with a third reviewer consulted as needed. Inter-observer reliability was assessed using Cohen’s kappa coefficient during both screening phases. Agreement between reviewers was strong (*κ* = 0.82 for title/abstract screening; κ = 0.87 for full-text screening), indicating high consistency in study selection. The study followed PRISMA (2020) guidelines, and reasons for exclusion at the full-text screening stage were documented. After screening 703 studies, 19 high-quality empirical studies were included in the synthesis.

### Data extraction and synthesis

A sample data extraction sheet was developed and piloted before use in the main analysis. Extracted data included author, year, journal, study aims, research design, sample details, main findings, and identified limitations. Using a narrative synthesis framework, findings were organized into four main domains: (1) prevalence and symptomatology of ODD and CD, (2) specific risk and protective factors, (3) neurobiological correlates, and (4) intervention efficacy. The method followed principles established by Popay et al. (2006) for integrating diverse types of research in public health reviews.

### Risk of bias assessment

Risk of bias was assessed using the JBI appraisal tool appropriate to each study’s methodology (cross-sectional, longitudinal, or intervention-based). The assessment focused on study design, sample selection, data collection, and statistical analysis. While most studies were methodologically sound, limitations included lack of blinding, use of convenience samples, and reliance on self-report questionnaires. Sensitivity analyses were conducted to assess the impact of excluding lower-quality studies on the results of the synthesis.

### Eligibility criteria

Included studies were (a) published in peer-reviewed journals between 2016 and 2025; (b) focused on ODD or CD in Chinese youth aged 6–18 years; (c) used DSM-IV-TR, DSM-5-TR, or ICD-10 criteria; and (d) collected data using scientific methods (quantitative, qualitative, or mixed-methods). Excluded were reviews, editorials, dissertations, grey literature, studies focusing on adults, or research lacking clear diagnostic or methodological rigor. Applying these criteria, 19 studies were included in the final qualitative synthesis. These comprised epidemiological studies, longitudinal analyses, clinical research, neurobiological investigations, and intervention evaluations, providing a comprehensive understanding of ODD and CD in Chinese youth.

### Data analysis

**Figure 1:**
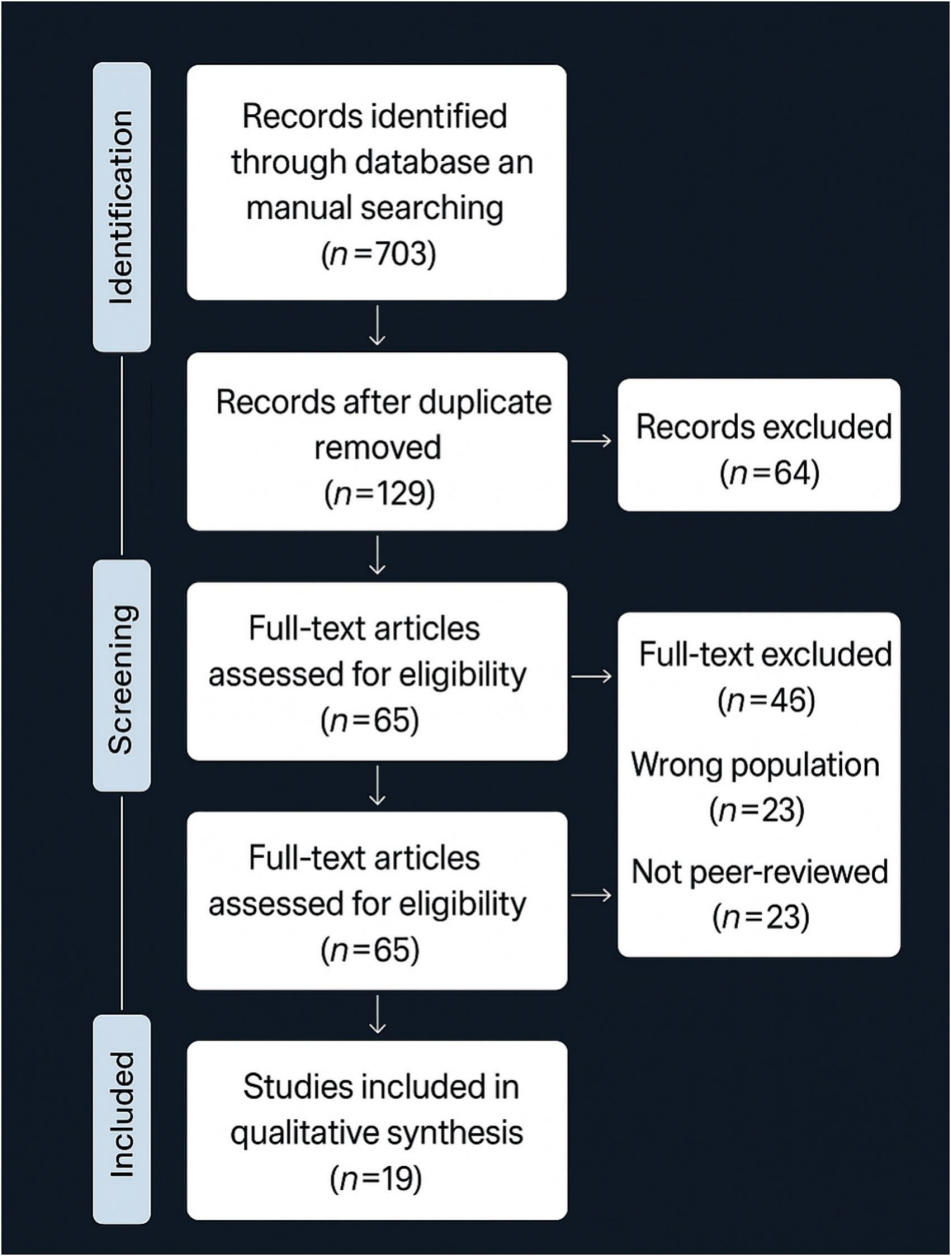
PRISMA diagram for study selection (adapted from Moher et al., 2009).

Data from included studies were analyzed using a narrative approach (Popay et al., 2006), integrating findings from quantitative, qualitative, and mixed-methods studies. Thematic analysis identified recurring concepts and patterns in symptomatology, family environment, cultural influences, neurobiological mechanisms, and intervention outcomes (Braun and Clarke, 2006). Each study was manually reviewed to extract key variables such as sample design, objectives, statistical methods, and main results. The number of studies by methodology, geographic region, and sample type was also recorded to provide an overview. Methodological quality and consistency were assessed to clarify differences among studies (Figure 1).

## Results

### Characteristics of included studies

The study of ODD and CD in Chinese child and adolescent groups is undergoing rigorous examinations, and 19 empirical studies were eligible to be included in the present review. Although they differ in design, scope, and population, all these studies contribute to a comprehensive understanding of disruptive behavior disorders in China. Research designs included cross-sectional studies (Li et al., 2016; Lin et al., 2016; Liu et al., 2017; Tang et al., 2017; Zhang et al., 2018; He et al., 2020; Zhang et al., 2023; Shen et al., 2018; Xie et al., 2024; Liu et al., 2025), longitudinal investigations (Liu et al., 2018; Wang et al., 2016; Jiang et al., 2020; Chen et al., 2022; Jiang and Lin, 2024), and experimental or quasi-experimental approaches (Lin and Gau, 2017; Wang et al., 2018; Zhu et al., 2021; Zhang et al., 2018; He et al., 2023a; He et al., 2023b). Three papers reported using a three-wave longitudinal design aimed at studying reciprocal relationships and developmental trajectories of ODD symptoms (Liu et al., 2018; Jiang et al., 2020; Chen et al., 2022), which can provide evidence of transactional processes between parental functioning and child behavioral outcomes. Some studies included mediation and moderation analyses to determine which factors influenced the outcomes of disruptive behavior, including parental depression, child emotion regulation, and family maltreatment (Li et al., 2016; Lin et al., 2016; Liu et al., 2017; Tang et al., 2017; He et al., 2020; Zhang et al., 2023; Lin et al., 2021; Lin et al., 2017; Lin et al., 2016). Together, this diversity in methods enhances the field by correlating epidemiological prevalence rates, psychosocial risk factors, neurocognitive correlates, and the effects of intervention within the context of cultural specificity.

The sample sizes across the nineteen studies varied, ranging from small experimental studies to large-scale nationwide surveys. The fewest participants were in the study by Zhu et al. (2021), which involved 98 children with ADHD, CD, and ADHD+CD, along with controls in a Stroop interference task, whereas Zhang et al. (2018) had 28 adolescents with CD and 28 controls in an fMRI study. On the other end of the spectrum, Liu et al. (2025) sampled 73,992 children in 12 provinces, and Li et al. (2022) evaluated 17,524 children in five provinces in a national psychiatric epidemiological survey, providing the largest data on prevalence. Most studies included school-aged children, some as young as 6 years old up to 14 years (e.g., Tang et al., 2017; Lin et al., 2016; Zhang et al., 2023; Shen et al., 2018), while others focused on delinquent and clinical samples of adolescents (e.g., Xie et al., 2024; Zhang et al., 2018). Some studies also involved parent–child pairs or parental sources to assess the family context (e.g., Liu et al., 2018; He et al., 2020; Chen et al., 2022; Liu et al., 2025; Liu et al., 2015; Liu et al., 2021; Liu et al., 2023).

About a third of studies specifically recruited children with ODD or ODD/CD diagnoses (Li et al., 2016; Lin et al., 2016; Tang et al., 2017; He et al., 2020; Liu et al., 2017), whereas others used broader sampling methods in schools or communities to capture more dimensions of symptoms (e.g., Zhang et al., 2023; Shen et al., 2018; He et al., 2023a; He et al., 2023b). Li et al. (2022), Wang et al. (2018), Liu et al. (2025), Masi et al. (2023), Mastnak (2022), Park et al. (2015), Tang et al. (2024), Wang et al. (2018), Wang et al. (2022), Zhang et al. (2023) and Zhou et al. (2024)) translated these findings to population-level prevalence by determining comorbidity and regional distribution patterns.

The majority of the studies defined ODD and CD according to DSM-IV-TR or DSM-5. The use of standardized assessment tools was high: Li et al. (2022) implemented the MINI-KID to ascertain psychiatric diagnoses; Shen et al. (2018) employed the Child Behavior Checklist (CBCL) to determine externalizing traits in left-behind children; Liu et al. (2025) applied the SNAP-IV for nationwide screening. These assessments were typically multi-informant, with reports provided by parents, teachers, and, in some instances, the children themselves (Li et al., 2016; Lin et al., 2016; Jiang et al., 2020; Chen et al., 2022). Some studies also used neurocognitive or structured tests to confirm diagnosis-related differences, including Lin and Gau (2017), who used comparative neuropsychological tests across ADHD, CD, ADHD+CD, and control groups, and Zhu et al. (2021), who utilized an experimental Stroop procedure.

Some research studies employed neuroimaging or biological approaches to analyze the mechanisms underlying disruptive disorders. Wang et al. (2016) explored gene–environment interactions and found that genetic vulnerability was exacerbated by harsh parenting and conflicts with peers. Using fMRI, Zhang et al. (2018) identified disturbed frontolimbic and default mode connectivity in adolescents with CD, providing evidence of neurobiological variances in aggression. Additional evidence of impaired inhibition, impulse control, and interference was reported in neurocognitive experiments conducted by Lin and Gau (2017) and Zhu et al. (2021), particularly in the comorbid ADHD+CD groups. Together, these varied methodologies reflect the range of strategies applied in Chinese research on ODD/CD, encompassing epidemiology, family dynamics, cognitive testing, and biological factors.

The analytic techniques used in the included studies were largely sophisticated, indicating growing methodological maturity in Chinese research on disruptive behavior disorders. The study by He et al. (2020) explored the interaction among parental depression, parent–child conflict, and child emotion regulation in predicting conduct problems among migrant children by applying multilevel moderated mediation modeling. Tang et al. (2017) used structural equation modeling on data collected from the perspective of parent–child dyads, showcasing that proximal family-level predictors surpass distal ones in expressing ODD indicators. Cross-lagged panel models were used in longitudinal research, where Liu et al. (2018) found bidirectional relationships between parenting stress and ODD symptoms, and Chen et al. (2022) observed reciprocal relations between parental emotional dysregulation and child ODD symptoms over three waves. Moreover, Zhang et al. (2023) conducted a network analysis to extrapolate the interconnections of ODD symptoms and family risks, identifying annoyance and vindictiveness as critical nodes linking symptom groups to family relations. Zhang et al. (2018) combined neuroimaging and data-driven analysis by comparing altered network connectivity between the frontolimbic and default mode networks in youths with CD, indicating the neural underpinnings of aggression (Table 1).

**Table 1:** Summary of included studies in SLR.

Authors	Years	Journal	Aims	Sample	Methodology	Findings	Limitations
Li et al.	2016	Journal of Interpersonal Violence	To explore associations among maltreatment, parent–child relationship, and emotional/behavioral problems in children with or without ODD.	259 ODD and 269 non-ODD children across three Chinese provinces	Cross-sectional, multi-informant survey	Emotional abuse predicted emotional problems; physical abuse predicted behavioral problems; parent–child relationship mediated effects in ODD children only.	Potential recall bias; no longitudinal design to assess causal direction.
Xie et al.	2024	Humanities and Social Sciences Communications	To determine the prevalence and risk factors of conduct disorder (CD) among juvenile delinquents in China.	545 male juvenile delinquents and 297 typically developing peers	Cross-sectional, structured assessments with standardized tools	58.7% of delinquents met CD criteria; deviant peer affiliation, low parental monitoring, and moral disengagement were major predictors.	Limited to male delinquents; lacks longitudinal perspective.
Liu et al	2018	Journal of Family Psychology	To examine the longitudinal relationship between parenting stress and ODD symptoms in children.	243 children with ODD and their parents	Three-wave longitudinal design using cross-lagged panel modeling	Bidirectional relationships observed; parenting stress predicted later ODD symptoms and vice versa, with gender differences in directionality of influence.	Long intervals between waves may reduce sensitivity to shorter-term changes
He et al	2021	Current Psychology	To examine how parental depression influences conduct problems in migrant children with ODD symptoms, via parent–child dynamics.	370 parents of migrant children with ODD from 10 elementary schools	Cross-sectional survey with moderated mediation analysis	Parental depression was linked to conduct problems, mediated by parent–child conflict and closeness, moderated by child emotion regulation ability.	Self-reported measures: migrant population may limit generalizability.
Zhang et al.	2023	Research on Child and Adolescent Psychopathology	To identify core family-level risk factors for ODD symptoms using network analysis.	718 Chinese migrant children aged 7–14 years	Cross-sectional, network analysis	“Annoy” and “vindictive” were central ODD symptoms; family cohesion, parent–child conflict, and parental depression were key bridge variables	Network model is correlational; causation not established.
Li et al.	2022	Journal of Child Psychology and Psychiatry	To estimate prevalence and comorbidity of mental disorders in a large sample of Chinese children and adolescents.	17,524 clinically assessed children aged 6–16 from 5 provinces in China	Two-stage national psychiatric epidemiological survey	17.5% prevalence of any disorder; ODD, CD, ADHD had high comorbidity; ODD and CD more prevalent in younger males and in developed areas.	National data lacks regional nuances; limited depth on ODD/CD-specific mechanisms
Lin et al.	2016	Child Abuse & Neglect	To investigate the relationship between child maltreatment and interpersonal relationships in children with ODD.	256 ODD children and their parents and teachers from Beijing, Shandong, and Yunnan	Cross-sectional, multi-informant survey	Emotional abuse linked to poor interpersonal functioning across parents, peers, and teachers; severe maltreatment predicted worse outcomes	Self-reports may introduce bias; no longitudinal data to establish directionality
Liu et al.	2017	Frontiers in Psychology	To explore family and individual-level predictors of depression among migrant children with ODD symptoms	368 migrant children with at least one ODD symptom	Cross-sectional, mediation and moderation analyses	Family maltreatment and negative automatic thoughts predicted depression; resilience and cohesion buffered these effects.	Non-random sample of migrants; focused only on depressive outcomes.
Tang et al.	2017	Frontiers in Psychology	To test multilevel family models for affective and behavioral symptoms of ODD in Chinese children.	80 father–child and 169 mother–child dyads from ODD families	Cross-sectional, structural equation modeling	Dyadic and individual-level family factors linked to affective symptoms; only proximal factors predicted behavioral ODD symptoms.	Focus limited to families with existing ODD cases; cross-sectional design limits causal interpretations.
Jiang et al.	2020	Child Psychiatry & Human Development	To examine the longitudinal effects of parental emotion dysregulation on children’s ODD symptoms via mediators like effortful control.	275 children and parents from Chinese primary schools	Three-wave longitudinal study	Parental emotion dysregulation indirectly increased ODD symptoms through impaired child effortful control; emotional regulation improved outcomes over time.	Attrition across waves; emotional regulation not directly observed but inferred through surveys.
Zhang et al.	2018	Neuropsychiatric Disease and Treatment	To examine functional brain connectivity in adolescents with conduct disorder using fMRI.	28 male adolescents with CD and 28 controls	Experimental neuroimaging (fMRI)	CD adolescents showed altered frontolimbic and DMN connectivity; abnormal coupling	Small sample size; only male participants; correlational fMRI findings.
Lin and Gau	2017	European Child & Adolescent Psychiatry	To compare neurocognitive function in children with ADHD, CD, ADHD+CD, and typically developing peers.	98 children from Taiwan, divided into four diagnostic groups	Clinical, comparative neuropsychological testing	ADHD+CD children had worse inhibition and emotional control than ADHD-only or CD-only; CD-only showed relatively intact cognitive performance.	Limited to Taiwanese sample; neuropsychological measures may not fully reflect real-world behavior.
Wang et al.	2018	Journal of the American Academy of Child Psychiatry	To assess whether ADHD medication reduces future risk of ODD and CD in a large Taiwanese cohort.	33,835 children diagnosed with ADHD from national health records	Population-level longitudinal cohort	ADHD medication adherence reduced risk of developing ODD by 53% and CD by 58%; dose-dependent effect observed.	Medication adherence measured via pharmacy refill data; no behavioral data from clinical settings.
Chen et al.	2022	Journal of Youth and Adolescence	To explore the longitudinal relationship between parent emotion dysregulation and children’s ODD symptoms across three waves.	275 parent–child dyads from urban and rural schools in China	Three-wave longitudinal design	Declines in parental emotional dysregulation predicted reductions in children’s ODD symptoms; relationship mediated by improved child ER.	Measures based on self-report; did not assess school-based moderators.
Zhu et al.	2021	Neuroscience Letters	To examine emotional interference control using the Stroop task in children with ADHD and comorbid CD.	98 children with ADHD, CD, ADHD+CD, and controls	Experimental design with Stroop task	ADHD+CD children had the worst interference control; ADHD-only group improved significantly with methylphenidate; CD mitigated cognitive gains.	Task may not generalize to daily functioning; medication effects not tracked over time.
Wang et al.	2016	Scientific Reports	To assess the interaction of genetic variants and environmental adversity in predicting ODD and CD symptoms.	453 Chinese children aged 7–15 from Beijing and Shandong	Longitudinal with genotyping and psychological surveys	G × E interaction found: genetic risk amplified by harsh parenting and peer conflict; emotional regulation moderated these effects.	Genetic effects small; complex interplay may need multivariate modeling in future studies.
He et al.	2023	Child Indicators Research	To evaluate the effectiveness of school-based social–emotional learning (SEL) intervention in reducing ODD symptoms in migrant children.	354 migrant children aged 7–12 in SEL and control groups	Quasi-experimental pre–post intervention design	SEL intervention reduced ODD symptoms and improved emotional regulation over 8 weeks.	Non-randomized design; short follow-up period.
Shen et al.	2018	Journal of Child and Family Studies	To assess how family cohesion and adaptability relate to behavioral problems in left-behind children with ODD symptoms.	328 left-behind children in rural China	Cross-sectional design using parent-reported measures	Lower family cohesion and adaptability were significantly associated with higher externalizing behavior scores including ODD symptoms.	Rural-only sample; left-behind context limits generalizability.
Liu et al.	2025	Psychiatry Research	To identify nationwide prevalence and symptom profile patterns of ADHD and its comorbidities, including ODD and CD.	73,992 children across 12 Chinese provinces	Cross-sectional, national mental health screening	Prevalence of ADHD was 6.7%; comorbid ODD in 32% and CD in 11% of ADHD cases; externalizing comorbidity linked to more school and family impairment.	Self-report and screen-based diagnosis; cross-sectional design precludes causal insights.

ODD = oppositional defiant disorder; CD = conduct disorder; ADHD = attention-deficit/hyperactivity disorder; CBCL = Child Behavior Checklist; MINI-KID = Mini International Neuropsychiatric Interview for Children; ER = emotion regulation; fMRI = functional magnetic resonance imaging; SEL = socioemotional learning; DMN = default mode network.

## Findings

### Prevalence and comorbidity patterns of ODD and CD in Chinese children and adolescents

Regarding the prevalence of ODD and CD in China, ten key studies based on surveys and clinical assessments were reviewed to determine how widespread these diagnoses are among children and adolescents: Li et al. (2022), Shen et al. (2018), Xie et al. (2024), Liu et al. (2025), Zhang et al. (2024), Lin and Gau (2017), Zhu et al. (2021), Chen et al. (2022), Lin et al. (2016), Wang et al. (2018), Li et al. (2023), and Li et al. (2018).

Li et al. (2022) conducted a national study with 73,992 participants aged 6–16 years across five provinces. Clinicians then interviewed children using the MINI-KID, identifying 17,524 for further inclusion. The overall prevalence of any psychiatric disorder was 17.5% (95% confidence interval of 17.2–18.0). ODD and CD were among the most common disorders and showed high comorbidity with ADHD and depression. Both ODD and CD were more common in younger children than in adolescents and more prevalent among boys than girls. Additionally, youth in more developed provinces had higher reported rates than those in less developed areas. No significant difference in suicide risk was found between rural and urban areas when accounting for socio-developmental status (χ^2^ (1, *N* = 71,929) = 1.4, *p* = 0.239).

Shen et al. (2018) provided regional data from 17,071 children in central Hunan. ODD prevalence was 2.98%, making it the second most common externalizing disorder after ADHD (4.96%). Among children with ADHD, 25.15% also had ODD and 18.18% had CD, indicating frequent comorbidity among disruptive behavior disorders. Over one-third of children with any psychiatric condition had at least one additional disorder, highlighting the complexity of youth psychopathology.

Xie et al. (2024) compared the prevalence of CD in juvenile delinquents and non-delinquent adolescents. In the delinquent group, 58.7% met the criteria for CD, and 90.94% of those diagnosed received the diagnosis before adulthood. There was no significant association between the number of CD diagnoses and criminal status, but those with criminal records exhibited higher aggression. The authors suggested that deviant peer influence was the strongest predictor of CD (structure coefficient = 0.85), followed by poor parental monitoring and lack of moral values, indicating both individual and social influences on CD.

Liu et al. (2025) analyzed data from 73,992 children to assess the prevalence of ADHD and its comorbidities. The prevalence of ADHD was 6.4%, and ODD/CD comorbidity was most common in the ADHD-Combined and ADHD-Hyperactive subtypes, affecting 58%. Misbehavior clustered more frequently in younger boys, while older girls were more likely to have anxiety disorders, suggesting age and gender differences in diagnostic patterns.

Zhang et al. (2024) used a cross-lagged panel network model (CLPN) to analyze data from 263 children aged 6–13 with ODD and/or ADHD. The study identified bidirectional relationships between ODD and ADHD symptoms, with “annoy” and “blame” acting as bridges to ADHD symptoms such as “interrupts/intrudes” and “close attention.”

Chen et al. (2022) studied the impact of ODD symptoms on social relationships in 275 parent–child dyads. Persistent ODD symptoms reduced emotional regulation abilities in both children and parents, indicating that disruptive behaviors affect the emotional stability of the entire family. Children with ADHD and ODD often experience a wide range of comorbid medical and psychiatric conditions. Beyond commonly co-occurring disorders such as anxiety, depression, learning disabilities, autism spectrum disorder, conduct disorder, sleep disturbances, and tic disorders, research also points to elevated rates of medical conditions including asthma, allergic rhinitis, atopic dermatitis, gastrointestinal issues, epilepsy, migraines, sleep apnea, iron deficiency, lead exposure, thyroid dysfunction, and PANDAS/PANS. Lin et al. (2016), for example, found that children with allergic conditions—such as asthma, atopic dermatitis, and allergic rhinitis—were more likely to have ADHD and ODD. While this single study did not establish causality, it supports a growing interest in shared biological mechanisms, such as those involving the gut–brain–immune axis. These findings underscore the importance of evaluating both psychological and physical health in children with disruptive behavior disorders.

Wang et al. (2018) reviewed the data of 33,835 Taiwanese youths with ADHD and observed that when medication was adhered to, the risk of developing ODD or CD became very small. Sensitivity analyses suggested that ADHD medication in the form of long-acting injectables (LAI) can help avert the exacerbation of externalizing behaviors. Nevertheless, medication is not the only component of successful treatment. There is increasing evidence in favor of multimodal strategies for children with comorbid ODD or CD in the context of ADHD. These strategies usually include a combination of stimulant or non-stimulant pharmacological treatment, parent training, school-based treatments, and in more severe cases, cognitive–behavioral therapy (CBT), social skills training, or multisystemic therapy. Although such interventions may be effective in alleviating symptoms and enhancing functioning, certain challenges, including cognitive or emotional regulation issues, can still remain untreated, highlighting the value of a personalized and holistic approach to care.

Overall, epidemiological studies suggest that ODD and CD in Chinese youth are highly prevalent, more so in boys than girls, and in younger children than older teens. These disorders are frequently comorbid with other psychiatric diagnoses (particularly ADHD and depression) and medical conditions, though more investigation is required. The complex interplay of individual, familial, and social factors is particularly noteworthy, underscoring the need for comprehensive assessment and intervention strategies. ADHD treatment adherence reduces the risk of developing ODD or CD; however, despite treatment, some issues (e.g., cognitive difficulties) can persist.

### Socioeconomic status, migration, and environmental stressors

Environmental, social, and economic challenges, including migration, may contribute to the persistence and exacerbation of ODD and CD symptoms among Chinese children. A review of seven studies, featuring Liu et al. (2017), He et al. (2020), Lin et al. (2016), Zhang et al. (2023), Liu et al. (2018), He et al. (2023a), He et al. (2023b), and Zhang et al. (2018), demonstrates that these disorders are influenced by societal factors that shape school involvement and children’s ability to cope with challenges.

Liu et al. (2017) studied 368 migrant children in China who had been diagnosed with ODD. In this context, “migrant” refers to children who had relocated within China—typically from rural to urban areas—often due to parental employment. These children, particularly those facing impoverished conditions such as family maltreatment and a lack of essential resources, were significantly more likely to experience depressive symptoms. The study found that negative thinking patterns mediated the effect of maltreatment on depression (*β* = 0.27, *p* < 0.001), while high resilience served as a protective factor, weakening this association (interaction term *β* = −0.19, *p* < 0.05). However, the study did not include a control group of non-migrant or non-ODD children, so findings should be interpreted with caution. Nonetheless, the results highlight the compounded vulnerabilities faced by migrant children with behavioral disorders—likely stemming from disrupted support systems and limited access to consistent education, healthcare, and peer networks.

He et al. (2020) also analyzed data from 370 migrant children and found that having depressed parents was linked to more conduct problems, mediated by parent–child conflicts (*β* = 0.31, *p* < 0.001). Those with poorer emotional control were more likely to be affected, underscoring that family stability and the child’s ability to regulate emotions interact during migration. In urban centers, migrants can encounter various challenges in accessing healthcare, fair treatment, and keeping up with their studies. Many studies agree that family situations affected by poverty or relocation disrupt consistency in parenting and emotional support for the child.

According to Lin et al. (2016), economic pressures within families were linked to emotional abuse and neglect. These conditions, whether individually or collectively, were also correlated with poor functioning among ODD-affected children in their relationships with parents, peers, and teachers. It was observed that such environmental problems diminished trust and willingness to help one another, both of which are important for maintaining healthy behavior.

According to Zhang et al. (2023), lower socioeconomic status or transitions associated with frequent moves contribute to conflict or lower cohesiveness in the family environment. The analysis revealed that both structural disadvantage and disturbed attachment primarily influenced these main symptoms. Moreover, Zhang et al. (2018) employed fMRI and machine learning to examine the brain structures of adolescents with CD and observed changes in the areas of the brain responsible for controlling emotions and impulses. These adolescents often came from backgrounds of hardship, indicating that ongoing psychological pressure may lead to alterations in brain development and contribute to behavioral risk.

The findings of these studies suggest that ODD and CD not only involve emotional issues but also reflect the influence of physical and environmental stressors. Negative economic conditions, frequent relocation, and other forms of social instability can heighten parenting stress and disrupt children’s emotional security, increasing the likelihood of behavioral problems such as defiance and aggression. To address these risks, support should include accessible and affordable medical care (e.g., mental health services, behavioral therapy, medication when needed) as well as targeted government policies—such as financial assistance programs, subsidized healthcare, housing support, and school-based mental health resources—to assist families navigating poverty and transition.

### Gender and developmental differences

A significant but not extensively studied aspect of these disorders is how their symptoms differ by gender and age group among Chinese youth. This theme brings together research from Liu et al. (2025), Shen et al. (2018), Zhang et al. (2023), Tang et al. (2017), and Xie et al. (2024), showing unique symptoms, increased prevalence, and age-related differences in these conditions for men and women. It appears that in younger children, males display more noticeable behavioral problems while girls exhibit less visible emotional difficulties that increase as they grow. Based on research by Liu et al. (2025), ODD/CD is primarily found in young boys, whereas girls are more prone to anxiety and depressive disorders as they get older. The results indicate that in childhood, boys are more likely than girls to experience ODD and CD because they develop impulsiveness earlier and have less behavioral control. In many cases, girls show signs of emotional distress when they are younger but switch to harmful behaviors towards others when they become adolescents, which makes it harder to recognize early.

Shen et al. (2018) reported that boys in their sample of 17,071 had more cases of ODD and CD. Although gender-based differences in CD symptoms decreased in adolescents, it could be that getting older or experiencing certain social factors gradually lowers the gap in behavior. Tang et al. (2017) further analyzed this developmental difference by identifying both effective and behavioral aspects of ODD. It was noted that boys were more likely to be compliant, whereas girls tended toward irritability and resentment, with both tendencies increasing when they reached middle childhood. Xie et al.’s (2024) forensic population study revealed that most teens with CD were male and that nearly all the diagnoses were adolescent-onset, suggesting that conduct problems often emerge around early puberty. Zhang et al. (2023) also pointed out that vindictiveness and irritability caused by ODD were influenced differently in boys and girls when exposed to conflict at home, suggesting that gender affects the way ODD symptoms manifest in different settings.

### School and peer influences

Where Chinese children spend their time at school and with their peers can help increase or prevent the development of ODD and CD. This theme is based on five studies: Zhang et al. (2023), Xie et al. (2024), Lin et al. (2016), He et al. (2023a), He et al. (2023b), and Jiang et al. (2020), which explore how teacher–student relationships, classroom climate, peer rejection, and belonging to a deviant peer group interact with basic behavioral risks. In reality, social–contextual factors aren’t just minor stressors; they tend to influence and control how externalizing symptoms appear and persist. Zhang et al. (2023) pointed out that few school connections, a higher level of teacher conflicts, and lack of engagement with schoolwork were main predictors of the “annoy” and “defy” symptoms in central ODD. Based on their study of 718 children, it was observed that where there were issues in teacher–student relationships, ODD symptoms appeared in groups, suggesting that how children interact in school is tied to their ODD symptoms. Lin et al. (2016) noted that when kids experienced both home abuse and rejection at school, their ODD was often more intense than that of children facing only one of these issues. The impact of peers can be especially significant during adolescence. Xie et al. (2024) found through their research that the strength of deviant peer affiliation surpassed all other factors, especially parental monitoring and self-regulation, as a predictor of conduct disorder in juvenile delinquents (structure coefficient = 0.85). The findings showed that more than half of delinquents met CD criteria and those with stronger bonds to deviant peers tended to be more aggressive, demonstrating that social influence is a main reason for antisocial acts.

The presence of supportive relationships at school can help protect students. He et al. (2023a, 2023b) discovered that when teachers interact positively with students with ODD, it helps these children manage their emotions better (*β* = 0.29, *p* < 0.01), which is related to fewer signs of ODD over time. These results imply that by providing consistent and empathetic support, school staff can help prevent risky student behavior. Jiang et al. (2020) showed that how children perceive their relationships with teachers and peers acts as a mediating factor connecting emotional dysregulation with later ODD symptoms. Together, these investigations highlight that both schools and peer groups can influence the development of ODD and CD. Implementing behavior management strategies and fostering a positive classroom environment can help children continue to learn and behave appropriately.

### Cultural influences and diagnostic challenges

The interpretation and treatment of ODD and CD in China are influenced by cultural beliefs, parenting styles, and the methods used to diagnose these conditions. This theme is explored in only a few research studies; nevertheless, significant insights can be found in the work of Lin et al. (2016), Tang et al. (2017), Wang et al. (2016), Liu et al. (2017), and He et al. (2020). These studies suggest that social pressure related to obeying authorities, respecting hierarchy, controlling emotions, and cultural honor complicates the accurate diagnosis of ODD and CD in China, often resulting in delayed diagnoses or misinterpreted symptoms. A key issue is that child misbehavior and emotional responses are not clearly understood within Confucian ideas, which value obedience, social order, and respect for authority figures. As Tang et al. (2017) state, boys are more likely to have their anger viewed as a disorder, while this is less common for girls due to traditional ideas about a woman’s role in being quiet and well-behaved. Similarly, children who try to avoid emotions or isolate themselves may exhibit mild symptoms of ODD that go unnoticed. The research found that irritability and resentment in Chinese parents were considered more likely to affect their relationship with the child, as such negative emotions are perceived as threatening the family’s sense of peace.

According to Lin et al. (2016), Chinese parents are more inclined to handle misbehavior through punishment, particularly when a child’s actions might reflect poorly on the family. As a result, the child may experience both neglect of their feelings and stricter punishments, which can lead to more frequent acting out. Cultural expectations for parenting may cause symptoms of ODD to be perceived as everyday behavior, preventing parents from seeking help. Additionally, the application of DSM-influenced diagnostic tools in China may not fully capture the behavioral patterns observed in the culture. Wang et al. (2016) and Liu et al. (2017) noted that parents were less likely to report issues related to emotional dysregulation because they viewed such behavior as indicative of weak moral values or because of the social stigma surrounding these issues. According to He et al. (2020), cultural barriers to seeking psychological help often meant that parents’ problems and conflicts went unrecognized, hindering the child’s access to early support. These findings highlight the necessity of incorporating Chinese cultural values and family dynamics into both diagnoses and treatment plans. When culture is overlooked, many children with ODD and CD may be missed during diagnosis and receive interventions that do not address their true needs.

## Discussion

The current systematic review exploring ODD and CD in Chinese children and adolescents finds that its results are sometimes similar and sometimes different from those observed internationally. A study conducted by Li et al. in China in 2022 discovered that approximately 17.5% of the population had diagnosed psychiatric disorders, indicating that ODD, CD, ADHD, and depression often co-occur. Results from this study are lower compared to global reviews. According to Mohammadi et al. (2021), the pooled global CD rate was 3.6%, but the risk was found to be significantly higher in Western Europe and North America than in both East and South Asia. Polanczyk et al. (2015) indicated that more children and youth in the United States and Canada have externalizing behavior disorders such as ODD and CD than in East Asian nations.

As reflected in the international literature, ADHD in Chinese children and adolescents is often associated with comorbid ODD or CD. Liu et al. (2025) indicated that the prevalence of comorbidity in the combined and hyperactive forms of ADHD reached about 58 percent. Based on population-level investigations, 30–50 percent of children diagnosed with ADHD fulfill the criteria for CD or ODD in international populations (Gnanavel et al., 2019). These numbers resonate with the Western clinical use of the term, where clinical practice guidelines suggest that comorbidity with ADHD can be as high as 90 percent (Canadian Paediatric Society, 2018). Similar trends are observed in South Korea; Kim et al. (2023) reported that the presence of ADHD and ODD is associated with more significant neuropsychological deficits compared to ADHD alone. Similarly, European epidemiology has reported higher co-occurrence: Tesli et al. (2024) found that children with CD had a 10-fold increased likelihood of comorbid ADHD. Resources in Australia suggest that 33 percent to 50 percent of children with ADHD also have ODD (ADHD Support Australia, 2016). According to South African data, children with ADHD exhibit notable ODD and CD symptoms, particularly in boys (Mphahlele et al., 2023).

Early irritability and emotional dysregulation are predictors of later psychopathology across cultures. The Canadian longitudinal study indicates that preschool irritability predicts internalizing and externalizing disorders in adolescence (Sorcher et al., 2022). In a similar vein, longitudinal data links early irritability in childhood to subsequent depressive symptoms in adolescence across various global populations (Leno et al., 2024). In conclusion, findings of high ADHD and ODD/CD comorbidity among Chinese children are reflected globally, spanning East Asia, Europe, Australia, and Africa, suggesting that ADHD serves as a transdiagnostic risk factor. Cross-cultural predictive validity has been demonstrated, with irritability emerging as a predictor of psychopathology. These convergences provide a rationale for global calls for early and integrated identification and culturally sensitive intervention approaches.

In Chinese families, issues such as stress from moving, emotional imbalance within the family, and strife between parents are major factors in ODD and CD (Lin et al., 2019; He et al., 2020). This approach is also found in Canadian and Australian literature. Elbagir et al. (2023) undertook a review of various regions and found that a lack of parental care, parental indifference, and poverty all predicted disruptive behavior disorders in children worldwide. Chinese studies, in particular, note that emotional alignment between parents and children, as well as family emotional control, can lead to the emergence of such symptoms. From a neurocognitive perspective, Chinese scientists (e.g., Jiang et al., 2016) noted that both weak executive functions and poor emotional regulation can help predict ODD or CD. According to Gidziela et al. (2023), similar results were found in meta-analyses, with poor inhibition and working memory observed in children from the United Kingdom and the United States. Nonetheless, Chinese studies suggest that mental health issues among older people are often shaped by factors such as unrecognized struggles or traumas passed down through family generations.

How a society values cultural norms can influence the recognition and diagnosis of ODD and CD. Chinese culture, which emphasizes emotional restraint, may lead some girls to avoid expressing symptoms or to delay receiving a diagnosis (Tang et al., 2017). In contrast, Western society is more likely to regard dissent and aggression as negative mental health issues. According to Hodgkins et al. (2012), American and Canadian psychologists tend to study learning from antisocial peers, while East Asian psychologists pay special attention to aggressive behavior and family dynamics. Overall, studies conducted in China are comparable to those in other parts of the world, particularly because they suggest that children with ODD or CD often also have ADHD and are more likely to encounter environmental risk factors. However, Chinese literature offers unique insights into how emotion and culture affect the manifestation of symptoms and their diagnosis within the family. This indicates that a more effective approach to diagnosing and treating disruptive behavior disorders should be culturally sensitive.

## Conclusion

This review summarizes the findings from research on ODD and CD among Chinese children and adolescents, emphasizing how sociocultural, family, brain, and environmental factors are involved in the problem. According to the study, high levels of behavioral disorders are associated with parental authority, emotional issues, social rejection, and difficulties in school settings. Such risks are common worldwide, but Confucian cultural expectations influence how symptoms are expressed and how parents respond, indicating the need for culturally specific approaches by clinicians and parents. Although findings from neurocognition suggest deficiencies in executive function for externalizing disorders, such studies are not well established in China. There is still a major need for longitudinal studies that involve treatment. This review highlights the importance of providing mental health care that aligns with the development and background of individuals, supported by recognized diagnostic methods and treatments from various systems. It creates a framework that can guide mental health policies across China and inspire further studies on behavioral disorders in schools and society.

### Limitations and recommendations

Despite the significant contribution to exploring the prevalence, comorbidity, and demographic trends of ODD and CD in Chinese children and adolescents, several limitations of this systematic review should be considered. First, most of the identified studies were cross-sectional in nature, complicating inferences about causal relationships between the observed and reported risk factors (i.e., family stress, peer rejection, and academic difficulties) and the development or maintenance of ODD or CD. Few longitudinal studies exist, limiting the ability to investigate longitudinal courses, cross-influence, and long-term effects.

Second, the sampling technique, diagnostic instruments, and outcome measures varied across studies, and this methodological heterogeneity limited the potential for meta-analysis and the applicability of results. Specifically, there was a wide range of diagnostic criteria and assessment instruments, with some relying primarily on parent and teacher reports. Such informants can be biased by cultural norms regarding acceptable behavior, which may reduce the validity of symptom profiles. The most common multi-informant method is still underutilized in Chinese ODD and CD research: different approaches, such as child self-reports and clinician-administered assessments.

Third, there is a possibility of publication bias and linguistic bias. Although this review captured both English and Chinese publications, there are still some Chinese journals that are underrepresented in international databases. This increases the likelihood that similar works, particularly those with null or negative findings, were not retrieved. Therefore, prevalence estimates and comorbidity distributions may be slightly overestimated.

Future research needs to focus on longitudinal designs that track risk and protective factors in development and comorbidity over time. Additionally, future studies should employ multi-wave longitudinal designs with shorter intervals between assessments, include multi-informant data from parents, teachers, and clinicians, and test bidirectional pathways between family stress, emotion regulation, and disruptive behavior. Such designs would allow for clearer developmental and causal inference. The use of diagnostic tools that are validated and culturally adapted for use in China is important for increased accuracy. There is also a need to increase investment in intervention studies, particularly randomized controlled trials of family-based and school-based interventions, to examine culturally appropriate strategies. Moreover, underserved groups (e.g., rural and migrant children) should be explicitly included in research to ensure the validity of findings across diverse demographic groups. Increasing school- and pediatric-based screening programs with culturally competent professionals will be essential in minimizing the turnaround time for diagnosing and providing access to timely care.

## Data Availability

The original contributions presented in the study are included in the article/supplementary material, further inquiries can be directed to the corresponding authors.
