# Correction: Prevalence, comorbidity, and demographic patterns of oppositional defiant disorder and conduct disorder in Chinese children and adolescents: a systematic review

**DOI:** 10.3389/fpsyg.2026.1794314

**Published:** 2026-02-09

**Authors:** S. Mudasser Shah, Ghada Saleh Alhudaithi, Ghada Saad Altalha, Chand Taneja, Fatimah Sayer Alharbi, Xiuyun Lin

**Affiliations:** 1 Institute of Developmental Psychology, Beijing Normal University, Beijing, China; 2 Self-Development Skills Department of common First Year Deanship, King Saud University, Riyadh, Saudi Arabia; 3 Department of Self-Development Skills, King Saud University, Riyadh, Saudi Arabia; 4 Department of Neuropsychology Program, Queen Alexandra Center for Children's Health, Victoria, BC, Canada; 5 Department of Health Sciences, College of Health and Rehabilitation Sciences, Princess Nourah Bint Abdulrahman University, Riyadh, Saudi Arabia; 6 Beijing Key Laboratory of Applied Experimental Psychology, Faculty of Psychology, Beijing Normal University, Beijing, China

**Keywords:** oppositional defiant disorder, conduct disorder, children, adolescents, China, prevalence

Author Fatimah Sayer Alharbi was erroneously assigned to affiliation Department of Psychology, College of Education and Human Development, Princess Nourah Bint Abdulrahman University, Riyadh, Saudi Arabia. This affiliation has now been removed for author Fatimah Sayer Alharbi.

The original version of this article has been updated.

